# Neuropsychological functions of verbal recall and psychomotor speed significantly affect pain tolerance

**DOI:** 10.1002/ejp.1437

**Published:** 2019-07-29

**Authors:** Henrik Børsting Jacobsen, Audun Stubhaug, Henrik Schirmer, Nils Inge Landrø, Tom Wilsgaard, Ellisiv Bøgeberg Mathiesen, Christopher Sivert Nielsen

**Affiliations:** ^1^ Department of Pain Management and Research, Division of Emergencies and Critical Care Oslo University Hospital Oslo Norway; ^2^ Norwegian National Advisory Unit on Neuropathic Pain, Division of Emergencies and Critical Care Oslo University Hospital Oslo Norway; ^3^ Institute of Clinical Medicine, Faculty of Medicine University of Oslo Oslo Norway; ^4^ Department of Cardiology Akershus University Hospital Lørenskog Norway; ^5^ Department of Clinical Medicine UiT The Arctic University of Norway Tromsø Norway; ^6^ Clinical Neuroscience Research Group, Department of Psychology University of Oslo Oslo Norway; ^7^ National Competence Centre for Complex Symptom Disorders St. Olavs University Hospital Trondheim Norway; ^8^ Department of Community Medicine UiT The Arctic University of Norway Tromsø Norway; ^9^ Department of Neurology University Hospital of North Norway Tromsø Norway; ^10^ Division of Ageing and Health Norwegian Institute of Public Health Oslo Norway

## Abstract

**Background:**

Effects from cognitive performance on pain tolerance have been documented, however, sample sizes are small and confounders often overlooked. We aimed to establish that performance on neuropsychological tests was associated with pain tolerance, controlling for salient confounders.

**Methods:**

This was a cross‐sectional study nested within the Tromsø‐6 survey. Neuropsychological test performance and the cold pressor test were investigated in 4,623 participants. Due to significant interaction with age, participants were divided into three age groups (<60, ≥60 to <70 and ≥70 years). Cox proportional hazard models assessed the relationship between neuropsychological tests and cold pressure pain tolerance, using hand‐withdrawal as event. The fully adjusted models controlled for sex, education, BMI, smoking status, exercise, systolic blood pressure, sleep problems and mental distress.

**Results:**

In the adjusted models, participants aged ≥70 years showed a decreased hazard of hand withdrawal of 18% (HR 0.82, 95% CI (0.73, 0.92) per standard deviation on immediate verbal recall, and a decreased hazard of 23% (HR 0.77, 95% CI (0.65, 0.08) per standard deviation on psychomotor speed. Participants aged ≥60 to <70 years had a significant decreased hazard of 11% (HR 0.89, 95% CI (0.80, 0.98) per standard deviation on immediate word recall. In participants aged <60 years, there was a decreased hazard of 14% (HR 0.86 95% CI: 0.76, 0.98), per standard deviation on psychomotor speed.

**Conclusion:**

Better performance on neuropsychological tests increased pain tolerance on the cold pressor test. These exposure effects were present in all age groups.

**Significance:**

This paper describes substantial associations between cognitive functioning and cold pressor tolerance in 4,623 participants. Reduced psychomotor speed and poor verbal recall gave greater odds for hand‐withdrawal on the cold pressor task. The associations were stronger in older participants, indicating an interaction with age.

## 1 INTRODUCTION

Pain is a complex phenomenon, which includes sensory‐discriminative, motivational‐affective and cognitive‐behavioral factors (Melzack & Casey, 1968; Turk & Melzack, 2011). Due to its complex nature, cognitive processing is required whenever pain is to be consciously registered.

Neuropsychological tests (or tasks) infer adequacy of cognitive processes through theories about the functioning of underlying neuronal correlates. Within pain research, both neuropsychological tests and underlying theory has been criticized for lack of reliability and delineation (Moriarty, McGuire, & Finn, 2011). Still, neuropsychological tasks are commonly used to investigate how a painful experience is correlated or associated with cognitive performance (Landrø et al., 2013). Subsequent data have led to a theory, stating that pain demands cognitive resources and therefore limits cognitive functioning due to interference with neural networks (Legrain et al., 2009; Moriarty et al., 2011). Such interference could explain cognitive dysfunction in individuals with persistent pain. This is a frequent and debilitating complaint, and therefore, it warrants extensive study.

Existing lines of research seem to indicate a bidirectional relationship, where experiencing pain is associated with lowered neuropsychological performance, and lowered neuropsychological test performance alters the experience of pain.

In studies of experimental pain tolerance there are data to suggest that inhibitory capacity (Oosterman, Dijkerman, Kessels, & Scherder, 2010) and visuospatial skills (Terrighena, Shao, & Lee, 2017) influence pain tolerance in healthy participants. Moreover, that reduced pain tolerance indicates reduced cognitive inhibition or cognitive control. Data also points to age playing an important role in the presence and/or severity of the changes in neurocognitive substrates and how they affect pain tolerance (Marouf et al., 2014).

Numerous studies have focused separately on age‐related changes in the pain experience or on age‐related changes in cognitive functioning (Oosterman & Veldhuijzen, 2016). However, little is known about the relationship between these two factors (Oosterman & Veldhuijzen, 2016). The available studies are few and conflicting, but in younger participants pain ratings appear inversely related to memory and executive function. In older adults, pain ratings are either not associated or increase with better neuropsychological test performance (Oosterman, Gibson, Pulles, & Veldhuijzen, 2013). Data from those with cognitive impairments due to ageing further supports the notion that pain processing appears to change with age (Defrin et al., 2015; Marouf et al., 2014).

There is, however, a palpable lack of studies investigating how age and neuropsychological test performance is related to the pain experience in larger samples (Berryman et al., 2013; Moriarty et al., 2011). Although numerous randomized studies on cognitive performance and experimental pain tolerance typically range from 20 to 50 participants (Oosterman et al., 2010, 2013; Santarcangelo et al., 2013; Terrighena et al., 2017). Such limited sample sizes present two significant problems.

First, the phenomenon reported might be due to large individual variation in low sample sizes. Second, a confounding variable might explain in part, or fully, the reported associations. To exemplify such confounding, experimental pain tolerance have been shown to be affected by sex (Alabas, Tashani, Tabasam, & Johnson, 2012), body mass index (BMI) (Price, Asenjo, Christou, Backman, & Schweinhardt, 2013), sleep problems (Lautenbacher, Kundermann, & Krieg, 2006), depression (Dickens, McGowan, & Dale, 2003), anxiety (Bruehl, Carlson, & McCubbin, 1992), blood pressure (Myers, Robinson, Riley, & Sheffield, 2001) and physical exercise (Meeus, Roussel, Truijen, & Nijs, 2010).

Thus, the primary aim of this study was to use data from an existing large‐scale study to establish or reject a link between the performance on neuropsychological tests and experimental pain in different age groups. We wanted to test the hypothesis that pain tolerance on the cold pressor test was associated with performance on three neuropsychological tests in different age groups, while controlling for sex, education, BMI, smoking status, exercise, systolic blood pressure, sleep problems mental distress, chronic pain and the use of analgesics.

## 2 MATERIALS AND METHODS

### 2.1 Setting and participants

The Tromsø study is a population‐based study with participants recruited from the municipality of Tromsø in Northern Norway. The study thus far consists of seven health surveys, carried out every sixth or seventh year since 1974. All the surveys include questionnaire data, sensory testing and clinical measurements. The aim has been to include large, representative samples of the Tromsø population, with invitation of whole birth cohorts and random samples. For a detailed description of the sampling procedure and selection choices, see Jacobsen and colleagues (Jacobsen, Eggen, Mathiesen, Wilsgaard, & Njølstad, 2012). The different surveys are referred to as Tromsø 1–Tromsø 7. The current investigation is a cross‐sectional design based on data from the Tromsø 6 survey (data collected from October 2007–December 2008).

The sample in Tromsø 6 was recruited as follows: a 10% random sample of individuals aged 30–39 (*n* = 1,056), all residents aged 40–42 and 60–87 (*n* = 12,578), a 40% random sample of inhabitants aged 43–59 (*n* = 5,787) and all subjects who had attended the second visit of Tromsø 4, but who were not already included in the three groups above (*n* = 341). Of the invited 19,762 individuals, 65.7% participated in the Tromsø 6 survey (*n* = 12,984). Three participants later withdrew consent, making the number of participants 12,981. Of these, 53.4% were women and age ranged from 30 to 87. Sampling procedures are detailed in multiple publications (Arntzen, Schirmer, Wilsgaard, & Mathiesen, 2011; Eggen, Mathiesen, Wilsgaard, Jacobsen, & Njolstad, 2013).

All participants in Tromsø 6 were asked to participate in the cold pressor test, but a pre‐defined subgroup of 7,307 participants (subjects older than 35 years of age) was also invited to attend cognitive testing. However, some were turned away due to capacity problems (lack of sufficient research staff to conduct assessments during peak hours, or when research staff was ill). The total pool of potential participants who attended at least one cognitive test was 5,797 participants. Of these, 4,472 participants had complete data on all cognitive tests and were suited for inclusion in the current study. Participants went through the cold pressor task and cognitive testing with a 1 week interval, first they performed the cold pressor task, then a week later they performed the cognitive tests.

### 2.2 Ethics

Tromsø 6 was approved by the Data Inspectorate of Norway and the present study was approved by the Regional Committee of Medical and Health Research Ethics, Northern Norway. The study complies with the Declaration of Helsinki, International Ethical Guidelines for Biomedical Research Involving Human Subjects and the International Guidelines for Ethical Review of Epidemiological Studies. Participation was voluntary. Each subject gave written informed consent prior to participation.

### 2.3 Dependent variable

#### 2.3.1 Cold pressor test

A 3°C circulating water bath (Julabo PF40‐HE, JULABO Labortechnik GmbH, Seelbach, Germany) connected to a 13 L external plexiglass container with a flow of 22 L/Min, was used in the cold pressor test.

The procedure began by having participants seated in a comfortable chair with instructions to relax. Then, participants were asked to submerge their dominant hand up to the wrist in the cold water, with instructions to continue until their pain tolerance was reached, or the full test was completed (106 s). If the test official was asked by the participant about when to withdraw, instructions given by the official were “to hold for as long as you are able”. The choice of 106 s was due to extensive pilot data indicating that only a very small percentage of participants withstanding cold pressor pain in 106 s, consequently withdrew their hand before reaching a pre‐specified 3‐min mark. When testing the number of subjects in the Tromsø 6 study, a full 3‐min test compared to 106 s would increase the cumulative workload with 142 hr on research technicians. Also we considered that longer exposure could represent a safety issue in this elderly population Therefore, the 106 s benchmark was chosen to increase the feasibility and minimize the risk. Pain tolerance was defined as the number of seconds submerged in those pulling their hand out of the water before reaching the full mark of 106 s. The time at which they pulled their hand out was then used as measure of pain tolerance and as the event in the Cox proportional hazards models. For illustration and figure purposes, participants were described as pain sensitive and pain tolerant based on whether or not they withdrew their hand from the cold water before 106 s.

#### 2.3.2 Neuropsychological tests

Cognitive function was assessed by three standardized tasks, chosen for their feasibility as screening tests in an epidemiological setting with a large number of participants. The chosen tests have been found to predict early cognitive decline in population‐based studies (Knopman et al., 2001; Palmer, Bäckman, Winblad, & Fratiglioni, 2003). In addition, the relationship between the ability to withstand experimental pain, cognitive functioning and how this could relate to differential cognitive load has been described in earlier publications (Eccleston, 1994). The tests chosen in the current study are classified as easy (recognition), moderate (immediate verbal recall) and taxing (psychomotor speed).

In the word recognition test (recognition) a list of words is presented, including the 12 nouns from the previous recall task, alongside 12 random distractor items. Here the participants receive 2 points for each word identified from the noun list (hits), giving a perfect score of 24, with a range from 0 to 24.

The 12‐word memory test (immediate verbal recall) is a test of immediate recall and working memory. Participants provide an immediate free recall of 12 nouns that were shown written on a board and also pronounced one at a time with a 5‐s interval. The participants then had 2 min to recall the words. One point was given for each word correctly recalled, giving a range from 0 to 12 points (Arntzen et al., 2011).

The Digit Symbol Coding test (psychomotor speed) is a part of the Wechsler adult intelligence scale (WAIS) (Wechsler, 2008) and is used to examine psychomotor speed. The digit symbol substitution task consists of rows containing small blank squares, each paired with a randomly assigned number from one to nine. Above these rows there was a printed key that paired each number with a different nonsense symbol. Following a practice trial on the first seven squares, the subjects were asked to consecutively fill in as many as possible of the blank spaces with the corresponding symbol within 90 s. Subjects were encouraged to perform the task as quickly and accurately as possibly without skipping numbers.

### 2.4 Covariates

The participants completed two self‐administered questionnaires (https://uit.no/forskning/forskningsgrupper/sub?p_document_xml:id=367276%26sub_xml:id=387084), both questionnaires were filled out before testing commenced and were given to the participants at the day of testing to provide self‐reported data about the selected covariates. The reason for having two questionnaires is that the first was filled out by every participant in the entire study population (12,981) and the latter only by those who attended cognitive testing and other more extensive tests.

*Age and sex* were obtained from the Norwegian Central Population Registry.

Information about *educational level* was obtained from Q1, where education was coded as 1 (Primary/secondary school and modern secondary school), 2 (Technical school, vocational school and 1–2 years senior high school), 3 (High school diploma), 4 (College/university less than 4 years), 5 (College/university 4 years or more) (Myrtveit et al., 2016).

Data on *smoking and exercise* were also obtained from items in the Q1 “Are you a daily smoker?” Smoking was coded as 0 (no), 1 (yes, previously), 2 (yes, currently). *Exercise* was obtained from asking “How often do you exercise?” and coded as 0 (Never), 1(less than once a week), 2 (Once a week), 3 (2–3 times a week), 4 (approximately every day) (Myrtveit et al., 2016).

*Systolic blood pressure* was recorded with an automatic device (Dinamap Vital Signs Monitor, Tampa, FL, USA) by specially trained personnel (Myrtveit et al., 2016).

Height and weight were measured in centimetres and kilograms, respectively. *Body mass index (BMI)* was calculated as weight in kilograms divided by the square of the height in meters (kg/m^2^) (Myrtveit et al., 2016).

*Symptoms of anxiety and depression* were assessed with the Hopkins Symptom Checklist‐ (HSCL‐) 10. Participants scoring ≥1.85 were classified as having high symptomatology of anxiety and depression (Derogatis, Lipman, Rickels, Uhlenhuth, & Covi, 1974).

*Sleep problems* was assessed by a single item: “Difficulty in falling asleep or staying asleep” and coded as 0 (Not bothered at all), 1 (A little bothered), 2 (Quite bothered), 3 (It bothers me a lot) (Myrtveit et al., 2016).

*Chronic pain* was assessed by the following question (yes/no):

“Do you have persistent or frequently recurring pain that has lasted for 3 months or more?” (Myrtveit et al., 2016).

The use of analgesics was assessed by the following item “How often have you used painkillers [with]/[without] prescription during the last four weeks?” and was coded as 0 (Not used), 1 (Less than every week), 2 (Every week, but not daily), 3 (Daily).

### 2.5 Statistical analyses

Demographical data from participants were presented using frequencies (dichotomous variables) or means and standard deviations (*SD*) where appropriate.

The analytic approach used Cox proportional hazard models to investigate the effect of recall, coding and recognition (exposure) on cold pressor tolerance (outcome). The results are presented with the neuropsychological tests, systolic blood pressure and BMI being Z‐transformed, giving scores a common standard of zero as mean and unity standard deviation, and thereby facilitating their interpretation. The value of the z‐score shows how many standard deviations the individual participants' result deviates from the mean. When a z‐score is 0, the test score is equal to the mean. When a z‐score increases by +1, this means that the result is equal to one standard deviation (*SD*) above the mean of the test score.

Cox proportional hazard models were used to estimate hazard ratios for hand withdrawal on the cold pressor test. One model was assessed for each of the three neuropsychological tests (recall, coding and recognition) controlling for gender, education, smoking, exercise, systolic blood pressure, insomnia, mental distress and body mass index. To investigate two‐way interactions with age, three independent Cox proportional hazard models were used that included age as a continuous variable alongside cross product terms between age and the three neuropsychological tests. When interaction was established, we performed subgroup analyses with three optimal age categories based on a graphical visualization using a histogram of participant age distribution (<60, ≥60 to <70 and ≥70). All covariates were added in the model simultaneously: education level (0–3), BMI (z‐transformed), smoking status (0–2), Sleep problems (0–3), Systolic blood pressure (z‐transformed), HSCL‐10 (<1.85 or >1.85), analgesics (0–3) and chronic pain (0 or 1). The proportional hazard assumption was assessed by graphical inspection of log minus log survival curves (per quartile for continuous variables).

All statistical tests were two‐sided using a significance level of 0.05. Statistical analyses were conducted with SPSS version 22.0 (IBM Corporation, Armonk, NY).

## 3 RESULTS

The median age of the 4,472 participants which completed cognitive testing was 62 years and 44.9% were men. The participants had a mean BMI of 27.2 (*SD* 4.2) and mean systolic blood pressure of 140.9 (*SD* 22.6). The participants mean scores on coding, recognition and recall are detailed in Table 1. Participant demographics are further detailed in Table 2.

**Table 1:** Performance on the neuropsychological tests by age groups. The Tromsø Study 2007–2008

	<60 years of age		≥60 to <70 years of age		≥70 years of age	
	No	Mean (*SD*)	No	Mean (*SD*)	No	Mean (*SD*)
Age, years	1,420	53.7 (4.1)	2,068	63.6 (3.0)	1,135	75.1 (4.0)
Verbal memory test^a^	1,418	7.2 (1.8)	2,062	6.7 (1.8)	1,129	5.6 (1.9)
Word recognition test^b^	1,407	20.7 (3.3)	2,051	19.6 (3.9)	1,107	18.4 (4.8)
Digit‐Symbol coding test^c^	1,395	46.9 (11.4)	2,024	40.0 (11.7)	1,086	28.5 (10.9)

Note

Table shows participants stratified on age and presented as group 1 (<60), 2 (≥60 to <70) and 3 (≥70).

*SD*, Standard deviation

a Scores are given as the number of correct words recalled (0–12).

b Scores are given as the number of correct words recognized from a list of words (0–24).

c Scores are given as the number of right symbols coded (0–96).

**Table 2:** Overview of demographic variables presented as frequency and percent for all participants who attended cognitive testing

Demographic variables	Group 1	Group 2	Group 3
	*N* (%)	*N* (%)	*N* (%)
Sex			
Men	756 (53)	1,172 (57)	590 (52)
Females	664 (47)	896 (43)	545 (48)
Smoking			
Never	310 (22)	357 (17)	149 (13)
Previous	647 (46)	988 (48)	563 (50)
Active	458 (32)	700 (34)	396 (35)
Education			
Primary/secondary school	286 (20)	703 (34)	542 (48)
Technical school and vocational school	391 (28)	571 (28)	302 (27)
High school diploma	119 (8)	115 (6)	48 (4)
College/university less than 4 years	299 (21)	317 (15)	135 (12)
College/university 4 years or more	314 (22)	344 (17)	79 (7)
Sleep problems			
Not bothered	853 (60)	1,217 (59)	616 (54)
A little bothered	402 (28)	600 (29)	330 (29)
Quite bothered	118 (8)	144 (7)	83 (7)
It bothers me a lot	29 (2)	52 (3)	40 (4)
Exercise			
Never	56 (4)	86 (4)	81 (7)
Less than once a week	239 (17)	310 (15)	126 (11)
Once a week	314 (22)	400 (19)	200 (18)
2–3 times a week	560 (39)	819 (40)	384 (34)
Approximately every day	241 (17)	408 (20)	256 (23)
Anxiety and depression			
High	1,265 (89)	1,847 (89)	947 (83)
Low	125 (9)	122 (6)	61 (5)
Chronic pain			
No	977 (69)	1,409 (68)	800 (71)
Yes	441 (31)	656 (32)	333 (29)
Analgesics			
Not used	1,198 (84)	1,726 (84)	878 (77)
Less than every week	89 (6)	125 (6)	78 (7)
Every week, but not daily	69 (5)	91 (4)	61 (5)
Daily	44 (3)	63 (3)	55 (5)

Note

Table shows the age groups presented as group 1 (<60), 2 (≥60 to <70) and 3 (≥70).

### 3.1 Testing exposure from recall, coding and recognition on cold pressor tolerance

Recognition did not have a significant main effect when including age as a continuous variable, thus the variable was excluded from further analyses.

In the age stratified analysis of the *first age group* (<60 years), there were no significant associations between immediate verbal recall and cold pressor tolerance. There was however a 14% decreased hazard of hand withdrawal from the water per standard deviation on the coding test (hazard ratio (HR) 0.86, 95% confidence interval (CI) (0.76, 0.98), *p* = 0.02), controlling for all covariates. Moreover, there was a 17% reduced hazard of participants' hand withdrawal if they performed one standard deviation above the mean (HR 0.83, 95% CI (0.69, 0.99), *p* = 0.002).

When testing our hypothesis in the *second age group* (≥60 to <70 years) the data showed a decreased hazard of 11% (HR 0.89, 95% CI (0.80, 0.98), *p* = 0.017) of pulling your hand out of the water per standard deviation on the immediate word recall test, when controlling for all covariates. Performance on the coding test did not significantly affect pain tolerance in this age group.

**Figure 1:**
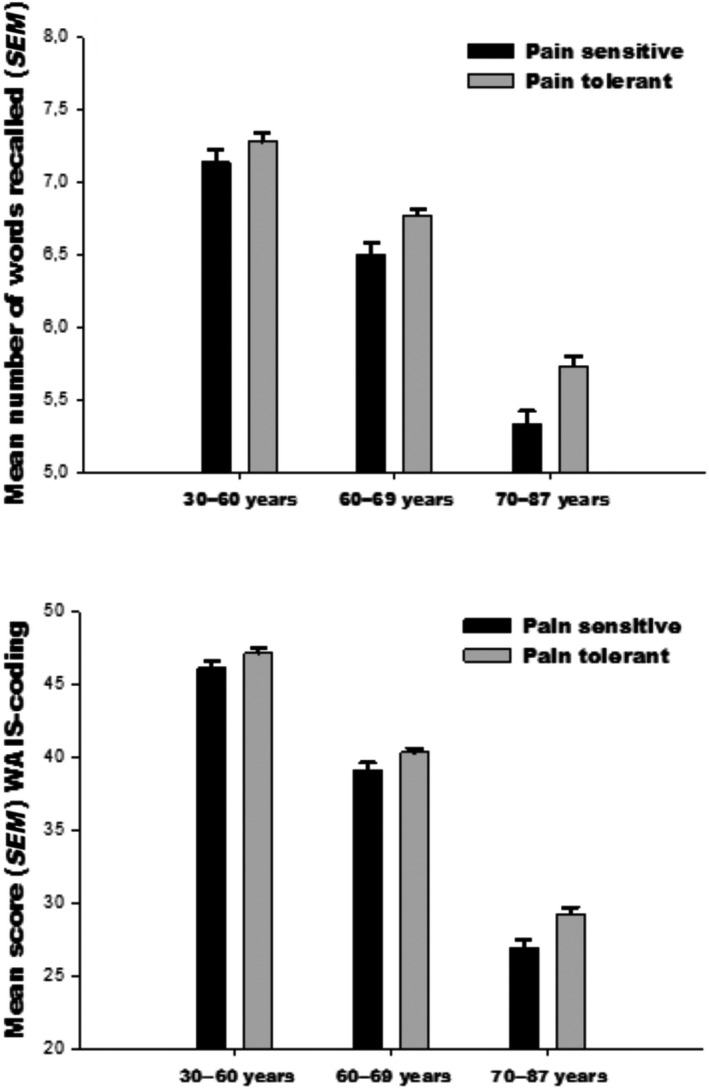
The participants were divided into two groups described as pain sensitive and pain tolerant based on whether or not they withdrew their hand from the cold water before 106 s. The mean scores on the two significant neuropsychological tests are presented with a standard error of the mean

When testing our hypothesis in the *third age group* (≥70 years), the data showed a decreased hazard of 18% (HR 0.82, 95% CI (0.73, 0.92), *p* < 0.001) per standard deviation on the recall test, when controlling for all covariates. The results also showed a decreased hazard of 23% (HR 0.77, 95% CI (0.65, 0.08), *p* = 0.002) per standard deviation on the coding test, controlling for all covariates. Detailed results from the Cox regression analysis of cold pressor tolerance are shown in Table 3. The raw mean score on coding and recall for the three age groups is presented with a standard error in Figure 1.

**Table 3:** The table displays exposure effects from neuropsychological test performance on cold pressor tolerance

Cogntive tests (age group)	*p* value	Hazard ratio (95% CI)
Coding (0)*	0.011	0.84 (0.74–0.96)
Coding (1)	0.484	0.96 (0.86–1.07)
Coding (2)	0.039	0.83 (0.69–0.99)
Recall (0)	0.157	0.93 (0.83–1.03)
Recall (1)*	0.021	0.89 (0.80–0.98)
Recall (2)*	0.015	0.85 (0.75–0.97)

Note

Event is defined as withdrawing your hand from the water, and hazard ratio is here presented with a 95% confidence interval. The age groups of 0, 1 and 2 corresponds to age groups of participants: <60 years of age (0); ≥60 to <70 years of age (1); ≥70 years of age (2). All exposure effects are controlled for sex, education, BMI, smoking status, exercise, systolic blood pressure, sleep problems and mental distress, chronic pain and the use of analgesics.

* *p* < 0.05

A sensitivity analysis to detect early onset dementia showed that removing 120 participants who scored less than 25 on the Mini‐Mental Status examination did not affect the results in any significant way. We further examined the potential effect of the cold pressor examiner on results and found no significant differences between research staff (Appendix).

### 3.2 Covariates

Of the covariates in the lowest age group (<60 years), education and severe insomnia were the only variables that significantly affected the hazard of pulling your hand out of the water when investigating the exposure effect from coding and recall. In this age group reporting a score of 3 “It bothers me a lot” increased the hazard of hand withdrawal with a 100% when investigating the recall task, with the exponent presenting a wide confidence interval (HR = 2.1 95% CI 1.7–3.8). There were only *n* = 29 participants reporting this score, which indicated a reason for the wide confidence interval.

Of the covariates in the second age group (≥60 to <70 years), systolic blood pressure, smoking and symptoms of anxiety and depression significantly affected the hazard of pulling your hand out of the water in both models.

In this age group being an active smoker had a significant effect on cold pressor tolerance in all three models tested, with the active smokers showing a reduced hazard for hand withdrawal.

Of the covariates in the third age group (>70 years) daily use of analgesics, exercise and severe insomnia were the only variables that significantly affected the hazard of pulling your hand out of the water in both models. In this age group reporting an equal to exercising once a week (2), or almost every day (4) reduced the hazard for hand withdrawal significantly. Moreover, using analgesics everyday (3) increased the risk of hand withdrawal with 84%. Further exposure effects from the different covariates are detailed in supplementary table 1.

## 4 DISCUSSION

This study points to a link between performance on tests of immediate verbal memory and psychomotor speed and experimental pain tolerance in a large sample. In accordance with our hypothesis there was a significant positive effect from performance on neuropsychological tests of immediate verbal memory and psychomotor speed on cold pressor tolerance in participants over 70 years of age. There was also a significant positive effect from the immediate verbal memory test in participants aged from 60 to 70 on cold pressor tolerance. In the youngest age group, there was a significant positive effect from performance on psychomotor speed on cold pressor pain tolerance.

In contrast to our results, a previous experimental study showed either a negative or no effect from neuropsychological test performance on pain tolerance in older adults (Oosterman et al., 2013). Others have even reported positive relationships of clinical pain ratings with executive function, but not memory or psychomotor speed, in both non‐demented institutionalized elderly (Oosterman, de Vries, Dijkerman, de Haan, & Scherder, 2009), and patients with dementia due to Alzheimer's disease (Scherder et al., 2008). The conflicting results in available literature give cause to doubt the relationship between neuropsychological tests and pain tolerance. However, we would argue that previous results in conflict with our findings could be a reflection of insufficient power due to small sample sizes. But, it could also be due to more comprehensive neuropsychological tests being included and/or differences in experimental pain stimulus (Oosterman et al., 2013).

The current results showed a positive effect from cognitive performance on pain tolerance. It could from these results be argued that the functioning of neural networks is both testable and have an effect on pain tolerance in elders. It is important to note that above 70 years of age is the age range at which declines in cognitive function and brain morphology can be expected. Although not tested directly, the effects on pain tolerance reported here may be related to grey and white matter loss in the prefrontal cortex (PFC), anterior cingulate cortex (ACC) and/or hippocampal regions due to ageing (Papp et al., 2014). Moreover, memory assessment through recall has demonstrated high levels of sensitivity and specificity to disorders causing memory dysfunction, such as amnestic mild cognitive impairment and Alzheimer's disease (Gavett & Horwitz, 2012), and we cannot exclude that such impairments exist in these participants, also affecting their pain tolerance. However, analyses of mini‐mental status indicated that only 120 of 4,492 participants scored lower than 25 points (Folstein, Folstein, & McHugh, 2019). Further analyses excluding these participants did not change the results, indicating that other underlying disease mechanisms such as early onset dementia or Alzheimer can be excluded as an explanation to the current findings.

It is important to note that while the MMSE is used in general practice, it is not to be considered a comprehensive assessment of cognitive decline. When compared to thorough diagnostics in a cohort with similar age profile as the participants' in this study, it had poor sensitivity (44%), but reasonable specificity.

(79%) in detecting mild cognitive impairment (de Jager, Schrijnemaekers, Honey, & Budge, 2009). Thus, there is still a reasonable possibility due to the age profile in these participants that they are experiencing cognitive decline, age related or otherwise, that is not picked up by the MMSE.

Our finding of test performance on recall having a significant effect on cold pressor pain tolerance in the participants aged 60–70 years of age also contradicts some previous findings, while it complements others. It is tempting to speculate that the current results are in line with findings of inhibition affecting pain tolerance (Zhou, Kemp, Després, Pebayle, & Dufour, 2015). While the neuropsychological tests did not include a specific test of inhibition, inhibition has been shown to activate a variety of PFC regions, and older adults have a greater activation in the regions that have been implicated in cognitive control and attentional interference (Zysset, Schroeter, Neumann, & von Cramon, 2007). This could entail that tests of immediate word recall is either subsumed or activates regions of the PFC which also involves inhibition (Conway & Engle, 1994).

The finding that education, exercise, symptoms of anxiety and depression, smoking and severe insomnia confound the relationship between neuropsychological test performance and cold pressor tolerance is in line with previous publications detailing effects on cold pressor tolerance from several factors (Defrin et al., 2015; Meeus et al., 2010; Myers, Robinson, Riley, & Sheffield, 2001; Sivertsen et al., 2015). The reported effects on exposure underline the importance of confounding variables when examining this relationship. It shows the necessity of a large sample size when performing experimental tests in order to firmly establish associations. It is important to note that while different covariates differ between the age groups, this is not the product of interaction, but reflects the fact that the age groups were independently analysed.

Of particular interest is the finding that education was the only variable to significantly affect pain tolerance in the youngest age group. If we are to speculate on why, education is often seen as a proxy for economic welfare, which has a strong relation to experienced life stress. Another explanation could be that the neuropsychological tests chosen were not sensitive in the younger group of participants, as they are specifically sensitive to age‐related changes of which education is a protective factor. Moreover, education has been showed to be a proxy of how well you are able to perform on tests independent of age, and this could very well be why it explains significant variance in the reported results (Ganguli et al., 2010).

The finding that severe sleep deficiency affects the relationship is also of interest given that insomnia could be a confounding variable, potentially affecting both the test performance and the cold pressor tolerance. Symptoms of insomnia has been associated with small to moderate impairments in memory and attention tasks (Fortier‐Brochu, Beaulieu‐Bonneau, Ivers, & Morin, 2012), as well as increased experimental pain sensitivity (Lautenbacher et al., 2006). In the recall and coding task, it was the youngest participants with severe insomnia that showed a significant effect on hand withdrawal. This is in line with a previous publication from the Tromsø 6 data showing a dose‐dependent relationship between insomnia severity and cold pressor tolerance (Sivertsen et al., 2015), but adds to those results that this relationship is reported only for those between 50 and 65 years of age. It is important to note that in a group with over 1,400 participants, only 29 participants reported severe problems, likely contributing to the reported effect.

Our finding that daily use of analgesics significantly decreasing cold pressor tolerance in the oldest age group are in line with previous findings from some of the current authors regarding analgesics and the cold pressor test (Samuelsen et al., 2017). These findings are somewhat contra‐intuitive, but indicate that the frequent use of analgesics is associated with a reduced pain tolerance in those over 70 years of age. Daily use of analgesics by older persons could then be a proxy for them being more pain sensitive in general. The endogenous opioid pathways recruited by analgesics are likely the same pathways involved in cold pressor tolerance, and the disruption of these would give higher pain sensitivity and reduced effect from analgesics (Samuelsen et al., 2017).

There is also a finding of smokers demonstrating higher pain tolerance when compared to non‐smokers. This has not been reported previously and is here reported in a large sample with a different age profile, contradicting studies using the same experimental pain model (Bagot, Wu, Cavallo, & Krishnan‐Sarin, 2017). If we were to speculate as to why this effect is present, it could be that it is related to findings of lowered heart rate variability in smokers, a factor that is strongly related to pain sensitivity and tolerance (Bruehl et al., 2018).

### 4.1 Limitations

A limitation of this study is the lack of a specific neuropsychological test of inhibition and a limited experimental pain stimulus. A second limitation is that this study had an observational design, and therefore, conclusions about causality cannot be drawn. Third, a longitudinal study is necessary to better examine the temporal relationships between chronic pain conditions, cognitive functioning and cold pressor pain. Moreover, in Norway, we have performed several population studies which show a similar level of self‐reported pain, depression and other relevant demographical variables as reported in the Tromsø study, but comparisons exceed the aim of the current study. However, 41% of Tromsø's population have university or college education, while the national average is 33.5%. A final limitation is that no adjustment was made to the model for unobserved person‐specific heterogeneity.

### 4.2 Conclusion

In this large sample of participants, better performance on neuropsychological tests of immediate verbal memory and psychomotor speed significantly increased pain tolerance on the cold pressor test. The reported effects from test performance interacted with participant age, but remained significant in all age groups when stratified. These findings point to the significance of cognitive functioning in the pain experience and contradict previous findings of an inverse relationship between cognitive performance and pain in older adults.

## CONFLICT OF INTEREST

None declared.

## APPENDIX 1

### Effects of examiners adjusted for on cognitive testing and cold pressor task

**Table:**

	B	SE	Wald	*df*	Sig.	HR	2.5% tile	97.5% tile
Recall adjusted analysis								
Age group 0	−0.076	0.054	2.000	1	0.157	0.927	0.834	1.030
Age group 1	−0.118	0.052	5.247	1	0.022	0.888	0.803	0.983
Age group 2	−0.162	0.066	5.994	1	0.014	0.850	0.747	0.968
Recall adjusted analysis + adjusted for examiner								
Age group 0	−0.088	0.054	2.625	1	0.105	0.916	0.823	1.019
Age group 1	−0.122	0.052	5.527	1	0.019	0.886	0.800	0.980
Age group 2	−0.162	0.067	5.907	1	0.015	0.850	0.746	0.969
Coding adjusted analysis								
Age group 0	−0.173	0.068	6.442	1	0.011	0.841	0.736	0.961
Age group 1	−0.038	0.057	0.449	1	0.503	0.963	0.862	1.076
Age group 2	−0.186	0.091	4.214	1	0.040	0.830	0.695	0.992
Coding adjusted analysis + adjusted for examiner								
Age group 0	−0.179	0.068	6.920	1	0.009	0.836	0.732	0.955
Age group 1	−0.028	0.057	0.241	1	0.624	0.972	0.869	1.088
Age group 2	−0.185	0.091	4.128	1	0.042	0.831	0.696	0.994
