# Supplementary material for: Neuropsychological functions of verbal recall and psychomotor speed significantly affect pain tolerance
Source: Eur J Pain. 2019 Jul 29;23(9):1608–18. doi: 10.1002/ejp.1437 (PMC6790685; doi:10.1002/ejp.1437)
Supplement: Supplementary file 1 [file EJP-23-1608-s001.docx]

Supplemental table 1. Full table showing the associations with the chosen event on the cold pressor task.

|  | **Variables** | ***P*** | **Hazard ratio  (CI 95 %)** |  | ***P*** | **Hazard ratio  (CI 95 %)** |  | ***P*** | **Hazard ratio  (CI 95 %)** |
| --- | --- | --- | --- | --- | --- | --- | --- | --- | --- |
| 0 | Sex | 0 | 1.82 (1.45 - 2.27) |  | 0 | 1.85 (1.48 - 2.31) |  | 0 | 1.87 (1.49 - 2.34) |
|  | Age | 0.619 | 0.99 (0.97 - 1.02) |  | 0.553 | 0.99 (0.97 - 1.02) |  | 0.266 | 0.99 (0.96 - 1.01) |
|  | Primary/secondary  school (Ref) | 0.02 | (Reference cat) |  | 0.01 | (Reference cat) |  | 0.011 | (Reference cat) |
|  | Technical school  vocational school | 0.028 | 1.38 (1.04 - 1.84) |  | 0.012 | 1.45 (1.09 - 1.94) |  | 0.008 | 1.49 (1.11 - 2.00) |
|  | High school diploma | 0.752 | 1.07 (0.71 - 1.60) |  | 0.598 | 1.12 (0.74 - 1.68) |  | 0.427 | 1.18 (0.78 - 1.78) |
|  | College/university  less than 4 years | 0.743 | 0.95 (0.68 - 1.32) |  | 0.951 | 0.99 (0.71 - 1.38) |  | 0.902 | 1.02 (0.73 - 1.44) |
|  | College/university  4 years or more | 0.579 | 0.91 (0.65 - 1.27) |  | 0.711 | 0.94 (0.67 - 1.32) |  | 0.912 | 0.98 (0.69 - 1.39) |
|  | Recall | 0.157 | 0.93 (0.83 - 1.03) |  |  |  |  |  |  |
|  | Coding |  |  |  |  |  |  | 0.011 | 0.84 (0.74 - 0.96) |
|  | BMI | 0.06 | 1.02 (1.00 - 1.05) |  | 0.046 | 1.03 (1.00 - 1.05) |  | 0.051 | 1.03 (1.00 - 1.05) |
|  | Never smoked (Ref) | 0.618 | (Reference cat) |  | 0.548 | (Reference cat) |  | 0.764 | (Reference cat) |
|  | Previous | 0.587 | 1.08 (0.83 - 1.40) |  | 0.628 | 1.07 (0.82 - 1.40) |  | 0.641 | 1.07 (0.81 - 1.40) |
|  | Active | 0.798 | 0.96 (0.72 - 1.29) |  | 0.675 | 0.94 (0.70 - 1.26) |  | 0.911 | 0.98 (0.73 - 1.33) |
|  | Never exercise (Ref) | 0.488 | (Reference cat) |  | 0.608 | (Reference cat) |  | 0.604 | (Reference cat) |
|  | Less than once a week | 0.235 | 0.71 (0.41 - 1.25) |  | 0.286 | 0.74 (0.42 - 1.29) |  | 0.309 | 0.75 (0.42 - 1.31) |
|  | Once a week | 0.817 | 0.94 (0.55 - 1.61) |  | 0.845 | 0.95 (0.55 - 1.63) |  | 0.905 | 0.97 (0.56 - 1.67) |
|  | 2-3 times a week | 0.695 | 0.90 (0.53 - 1.53) |  | 0.709 | 0.90 (0.53 - 1.53) |  | 0.777 | 0.93 (0.55 - 1.57) |
|  | Approximately every day | 0.51 | 0.83 (0.47 - 1.46) |  | 0.573 | 0.85 (0.48 - 1.50) |  | 0.641 | 0.87 (0.50 - 1.54) |
|  | Systolic blood pressure | 0.265 | 1.00 (0.99 - 1.00) |  | 0.266 | 1.00 (0.99 - 1.00) |  | 0.212 | 1.00 (0.99 - 1.00) |
|  | Not bothered by sleep problems (Ref) | 0.092 | (Reference cat) |  | 0.202 | (Reference cat) |  | 0.086 | (Reference cat) |
|  | A little bothered | 0.769 | 1.04 (0.82 - 1.31) |  | 0.899 | 1.02 (0.80 - 1.29) |  | 0.712 | 1.05 (0.83 - 1.33) |
|  | Quite bothered | 0.45 | 1.15 (0.80 - 1.67) |  | 0.501 | 1.14 (0.78 - 1.66) |  | 0.451 | 1.16 (0.79 - 1.68) |
|  | It bothers me a lot | 0.012 | 2.09 (1.17 - 3.71) |  | 0.036 | 1.89 (1.04 - 3.42) |  | 0.011 | 2.12 (1.19 - 3.78) |
|  | High anxiety and depression | 0.274 | 1.22 (0.86 - 1.74) |  | 0.147 | 1.30 (0.91 - 1.86) |  | 0.332 | 1.20 (0.83 - 1.72) |
|  | Chronic pain | 0.617 | 1.06 (0.84 - 1.35) |  | 0.685 | 1.05 (0.83 - 1.33) |  | 0.932 | 1.01 (0.79 - 1.29) |
|  | Do not use analgesics (Ref) | 0.701 | (Reference cat) |  | 0.743 | (Reference cat) |  | 0.659 | (Reference cat) |
|  | Less than every week | 0.602 | 0.90 (0.59 - 1.35) |  | 0.63 | 0.90 (0.60 - 1.36) |  | 0.83 | 0.96 (0.64 - 1.44) |
|  | Every week. but not daily | 0.919 | 1.02 (0.65 - 1.62) |  | 0.945 | 1.02 (0.64 - 1.61) |  | 0.704 | 1.09 (0.69 - 1.73) |
|  | Daily | 0.314 | 1.30 (0.78 - 2.16) |  | 0.346 | 1.28 (0.77 - 2.13) |  | 0.234 | 1.36 (0.82 - 2.27) |
|  |  |  |  |  |  |  |  |  |  |
| 1 | Sex | 0 | 1.71 (1.40 - 2.09) |  | 0 | 1.67 (1.37 - 2.03) |  | 0 | 1.68 (1.37 - 2.06) |
|  | Age | 0.07 | 1.03 (1.00 - 1.06) |  | 0.094 | 1.03 (1.00 - 1.06) |  | 0.174 | 1.02 (0.99 - 1.05) |
|  | Primary/secondary  school (Ref) | 0.798 | (Reference cat) |  | 0.562 | (Reference cat) |  | 0.736 | (Reference cat) |
|  | Technical school  vocational school | 0.841 | 0.98 (0.79 - 1.22) |  | 0.811 | 0.97 (0.78 - 1.21) |  | 0.867 | 0.98 (0.78 - 1.23) |
|  | High school diploma | 0.756 | 0.94 (0.63 - 1.40) |  | 0.65 | 0.91 (0.61 - 1.36) |  | 0.751 | 0.94 (0.62 - 1.41) |
|  | College/university  less than 4 years | 0.266 | 0.85 (0.63 - 1.13) |  | 0.16 | 0.81 (0.61 - 1.09) |  | 0.274 | 0.85 (0.63 - 1.14) |
|  | College/university  4 years or more | 0.387 | 0.88 (0.66 - 1.18) |  | 0.217 | 0.84 (0.63 - 1.11) |  | 0.278 | 0.85 (0.63 - 1.14) |
|  | Recall | 0.021 | 0.89 (0.80 - 0.98) |  |  |  |  |  |  |
|  | Coding |  |  |  |  |  |  | 0.484 | 0.96 (0.86 - 1.07) |
|  | BMI | 0.513 | 1.01 (0.99 - 1.03) |  | 0.425 | 1.01 (0.99 - 1.03) |  | 0.522 | 1.01 (0.99 - 1.03) |
|  | Never smoked (Ref) | 0.007 | (Reference cat) |  | 0.01 | (Reference cat) |  | 0.013 | (Reference cat) |
|  | Previous | 0.146 | 0.84 (0.66 - 1.06) |  | 0.181 | 0.85 (0.67 - 1.08) |  | 0.245 | 0.86 (0.68 - 1.11) |
|  | Active | 0.002 | 0.66 (0.51 - 0.86) |  | 0.003 | 0.67 (0.51 - 0.88) |  | 0.005 | 0.68 (0.52 - 0.89) |
|  | Never exercise (Ref) | 0.889 | (Reference cat) |  | 0.927 | (Reference cat) |  | 0.879 | (Reference cat) |
|  | Less than once a week | 0.689 | 1.10 (0.69 - 1.75) |  | 0.704 | 1.10 (0.68 - 1.77) |  | 0.623 | 1.13 (0.70 - 1.83) |
|  | Once a week | 0.847 | 1.05 (0.66 - 1.67) |  | 0.786 | 1.07 (0.67 - 1.71) |  | 0.811 | 1.06 (0.65 - 1.72) |
|  | 2-3 times a week | 0.77 | 1.07 (0.69 - 1.67) |  | 0.808 | 1.06 (0.67 - 1.66) |  | 0.756 | 1.08 (0.68 - 1.71) |
|  | Approximately every day | 0.843 | 0.95 (0.60 - 1.53) |  | 0.884 | 0.97 (0.60 - 1.56) |  | 0.891 | 0.97 (0.59 - 1.58) |
|  | Systolic blood pressure | 0.021 | 1.00 (0.99 - 1.00) |  | 0.017 | 1.00 (0.99 - 1.00) |  | 0.018 | 1.00 (0.99 - 1.00) |
|  | Not bothered by  sleep problems (Ref) | 0.539 | (Reference cat) |  | 0.579 | (Reference cat) |  | 0.573 | (Reference cat) |
|  | A little bothered | 0.454 | 1.08 (0.88 - 1.32) |  | 0.42 | 1.09 (0.89 - 1.33) |  | 0.537 | 1.07 (0.87 - 1.31) |
|  | Quite bothered | 0.424 | 0.86 (0.60 - 1.24) |  | 0.556 | 0.90 (0.62 - 1.29) |  | 0.478 | 0.88 (0.61 - 1.26) |
|  | It bothers me a lot | 0.549 | 0.85 (0.50 - 1.45) |  | 0.535 | 0.84 (0.49 - 1.44) |  | 0.432 | 0.80 (0.46 - 1.39) |
|  | High anxiety  and depression | 0 | 1.93 (1.40 - 2.66) |  | 0 | 1.88 (1.36 - 2.60) |  | 0 | 1.94 (1.40 - 2.69) |
|  | Chronic pain | 0.102 | 1.18 (0.97 - 1.44) |  | 0.081 | 1.20 (0.98 - 1.46) |  | 0.121 | 1.18 (0.96 - 1.44) |
|  | Do not use analgesics (Ref) | 0.53 | (Reference cat) |  | 0.523 | (Reference cat) |  | 0.483 | (Reference cat) |
|  | Less than every week | 0.602 | 0.91 (0.64 - 1.30) |  | 0.548 | 0.90 (0.63 - 1.28) |  | 0.566 | 0.90 (0.63 - 1.29) |
|  | Every week. but not daily | 0.205 | 1.29 (0.87 - 1.90) |  | 0.211 | 1.28 (0.87 - 1.89) |  | 0.188 | 1.30 (0.88 - 1.92) |
|  | Daily | 0.62 | 1.13 (0.71 - 1.80) |  | 0.666 | 1.11 (0.69 - 1.77) |  | 0.593 | 1.14 (0.71 - 1.82) |
|  |  |  |  |  |  |  |  |  |  |
| 2 | Sex | 0 | 1.78 (1.33 - 2.37) |  | 0 | 1.68 (1.26 - 2.24) |  | 0 | 1.71 (1.28 - 2.30) |
|  | Age | 0.231 | 1.02 (0.99 - 1.05) |  | 0.068 | 1.03 (1.00 - 1.07) |  | 0.132 | 1.03 (0.99 - 1.06) |
|  | Primary/secondary  school (Ref) | 0.366 | (Reference cat) |  | 0.283 | (Reference cat) |  | 0.459 | (Reference cat) |
|  | Technical school.  vocational school | 0.756 | 0.95 (0.71 - 1.28) |  | 0.527 | 0.91 (0.67 - 1.22) |  | 0.881 | 1.02 (0.76 - 1.39) |
|  | High school diploma | 0.493 | 0.80 (0.43 - 1.50) |  | 0.33 | 0.72 (0.38 - 1.39) |  | 0.735 | 0.90 (0.47 - 1.71) |
|  | College/university  less than 4 years | 0.046 | 0.62 (0.39 - 0.99) |  | 0.035 | 0.61 (0.38 - 0.97) |  | 0.081 | 0.66 (0.41 - 1.05) |
|  | College/university  4 years or more | 0.672 | 0.89 (0.53 - 1.51) |  | 0.589 | 0.87 (0.52 - 1.46) |  | 0.989 | 1.00 (0.58 - 1.72) |
|  | Recall | 0.015 | 0.85 (0.75 - 0.97) |  |  |  |  |  |  |
|  | Coding |  |  |  |  |  |  | 0.039 | 0.83 (0.69 - 0.99) |
|  | BMI | 0.87 | 1.00 (0.97 - 1.04) |  | 0.824 | 1.00 (0.97 - 1.04) |  | 0.718 | 1.01 (0.97 - 1.04) |
|  | Never smoked (Ref) | 0.203 | (Reference cat) |  | 0.239 | (Reference cat) |  | 0.349 | (Reference cat) |
|  | Previous | 0.71 | 0.93 (0.64 - 1.36) |  | 0.743 | 0.94 (0.63 - 1.39) |  | 0.737 | 0.94 (0.64 - 1.38) |
|  | Active | 0.142 | 0.74 (0.50 - 1.10) |  | 0.177 | 0.75 (0.50 - 1.14) |  | 0.227 | 0.78 (0.52 - 1.17) |
|  | Never exercise (Ref) | 0.151 | (Reference cat) |  | 0.118 | (Reference cat) |  | 0.083 | (Reference cat) |
|  | Less than once a week | 0.603 | 0.87 (0.51 - 1.48) |  | 0.617 | 0.87 (0.51 - 1.49) |  | 0.301 | 0.75 (0.44 - 1.29) |
|  | Once a week | 0.063 | 0.63 (0.39 - 1.03) |  | 0.067 | 0.63 (0.39 - 1.03) |  | 0.028 | 0.58 (0.35 - 0.94) |
|  | 2-3 times a week | 0.048 | 0.63 (0.40 - 1.00) |  | 0.033 | 0.60 (0.38 - 0.96) |  | 0.019 | 0.58 (0.37 - 0.91) |
|  | Approximately every day | 0.053 | 0.61 (0.37 - 1.01) |  | 0.055 | 0.61 (0.37 - 1.01) |  | 0.013 | 0.53 (0.32 - 0.88) |
|  | Systolic blood pressure | 0.073 | 1.00 (0.99 - 1.00) |  | 0.077 | 1.00 (0.99 - 1.00) |  | 0.077 | 1.00 (0.99 - 1.00) |
|  | Not bothered by sleep problems (Ref) | 0.524 | (Reference cat) |  | 0.621 | (Reference cat) |  | 0.616 | (Reference cat) |
|  | A little bothered | 0.898 | 1.02 (0.77 - 1.35) |  | 0.88 | 1.02 (0.77 - 1.36) |  | 0.989 | 1.00 (0.75 - 1.33) |
|  | Quite bothered | 0.39 | 1.23 (0.76 - 1.99) |  | 0.58 | 1.15 (0.71 - 1.86) |  | 0.46 | 1.20 (0.74 - 1.93) |
|  | It bothers me a lot | 0.161 | 1.63 (0.82 - 3.23) |  | 0.191 | 1.58 (0.80 - 3.12) |  | 0.222 | 1.53 (0.77 - 3.04) |
|  | High anxiety  and depression | 0.596 | 0.85 (0.47 - 1.55) |  | 0.884 | 0.96 (0.53 - 1.72) |  | 0.597 | 0.85 (0.47 - 1.55) |
|  | Chronic pain | 0.082 | 1.29 (0.97 - 1.73) |  | 0.05 | 1.34 (1.00 - 1.79) |  | 0.049 | 1.34 (1.00 - 1.79) |
|  | Do not use analgesics (Ref) | 0.019 | (Reference cat) |  | 0.021 | (Reference cat) |  | 0.009 | (Reference cat) |
|  | Less than every week | 0.395 | 1.22 (0.77 - 1.94) |  | 0.41 | 1.21 (0.77 - 1.92) |  | 0.261 | 1.30 (0.82 - 2.07) |
|  | Every week. but not daily | 0.154 | 0.64 (0.35 - 1.18) |  | 0.123 | 0.62 (0.33 - 1.14) |  | 0.134 | 0.63 (0.34 - 1.16) |
|  | Daily | 0.013 | 1.76 (1.13 - 2.75) |  | 0.019 | 1.71 (1.09 - 2.68) |  | 0.009 | 1.84 (1.17 - 2.90) |
